# Comparative evaluation of viability PCR reagents highlights the superior efficacy of PMAxx azo dye for bacterial and viral viability discrimination using real-time and digital PCR

**DOI:** 10.1128/spectrum.04002-25

**Published:** 2026-03-11

**Authors:** Tiwawan Hongsibsam, Jarunee Prasertsopon, Pilaiwan Siripurkpong, Nattamon Niyomdecha

**Affiliations:** 1 Graduate Program in Medical Technology, Faculty of Allied Health Sciences, Thammasat University 37698, Pathum Thani, Thailand; 2 Department of Medical Technology and Clinical Pathology, Jainad Narendra Hospital 432707 , Chainat, Thailand; 3 Center for Research Innovation and Biomedical Informatics, Faculty of Medical Technology, Mahidol University 26685, Nakhon Pathom, Thailand; 4 Department of Medical Technology, Faculty of Allied Health Sciences, Thammasat University 37698, Pathum Thani, Thailand; Connecticut Agricultural Experiment Station, New Haven, Connecticut, USA

**Keywords:** viability PCR (vPCR), PMAxx, EMA, PtCl₄, digital PCR, pathogen detection

## Abstract

Viability PCR (vPCR) distinguishes viable from nonviable microorganisms by suppressing amplification from structurally compromised cells or virions. This study compared three viability reagents—ethidium monoazide (EMA), propidium monoazide derivative (PMAxx), and platinum (IV) chloride (PtCl₄)—for differentiating viable and heat-inactivated *Staphylococcus aureus* (*S. aureus*), *Escherichia coli* (*E. coli*), human coronavirus OC43 (HCoV-OC43), and Enterovirus A71 (EV-A71) using real-time and digital PCR. EMA and PtCl₄ showed limited or inconsistent suppression of dead-cell or dead-virion signals, whereas PMAxx consistently demonstrated superior performance. Under optimal vPCR treatment conditions—100 µM PMAxx for *S. aureus*, 25 µM PMAxx with 0.01% sodium deoxycholate (DC) for *E. coli*, and 200 µM PMAxx for both HCoV-OC43 and EV-A71—the assays produced clear live–dead discrimination, with the highest ΔCq values reaching approximately 12 cycles in the most responsive organism–condition combinations. PMAxx completely suppressed amplification from heat-inactivated *S. aureus* (10⁶ CFU/100 µL) and EV-A71 (10² PFU/100 µL) while preserving signals from viable organisms, with concordant results across PCR platforms. Applying these optimized conditions to spiked matrices (milk, vegetable wash water, and nasopharyngeal transport medium) reduced ΔCq separation relative to culture suspensions, indicating matrix-associated interference, yet PMAxx maintained effective viability discrimination in most scenarios. This comparative analysis, with digital PCR serving as confirmatory evidence in representative high-contrast conditions, demonstrates that PMAxx provides the most reliable viability discrimination among the reagents evaluated and may be applicable to broader microbial viability studies and environmental surveillance contexts.

**IMPORTANCE:**

Molecular diagnostic assays are indispensable for rapid pathogen detection; however, their inability to distinguish viable from nonviable microorganisms can result in an overestimation of infection risk. The vPCR approach overcomes this limitation by combining selective nucleic acid-intercalating reagents with amplification-based detection. In this study, we evaluated EMA, PMAxx, and PtCl₄ across four representative pathogens—*S*. *aureus*, *E. coli*, HCoV-OC43, and EV-A71—using both real-time and digital PCR platforms. The results reveal that PMAxx provides superior performance in eliminating false-positive signals from nonviable cells and virions while preserving amplification from viable targets. The incorporation of digital PCR further enhances quantitative accuracy and absolute measurement of pathogen viability. These findings underscore the translational potential of PMAxx-based vPCR as a practical and robust strategy for improving molecular diagnostics, monitoring environmental contamination, and strengthening public health surveillance.

## INTRODUCTION

Molecular assays have emerged as powerful diagnostic tools, outperforming the gold-standard culture method in terms of detection speed, sensitivity, and specificity (1). However, a critical limitation is their inability to distinguish between viable and nonviable organisms, which may lead to an overestimation of microbial risk. DNA can persist in the environment long after cell death, and residual nucleic acids may resist degradation even under rigorous treatments, such as autoclaving at 121°C for 15 min (2). As only viable pathogens retain the ability to replicate and cause infection (3), distinguishing live from dead cells is essential for accurate hazard assessment, reliable surveillance, and robust public health risk evaluation (4).

To address this limitation, viability PCR (vPCR) has been developed through the use of nucleic acid-intercalating azo dyes, such as propidium monoazide (PMA), its optimized derivative PMAxx, and ethidium monoazide (EMA). These compounds selectively penetrate compromised cell membranes or disrupted viral capsids of nonviable microorganisms, where they covalently bind to DNA or RNA and effectively block subsequent amplification (5, 6). Recent studies indicate that platinum compounds, including platinum (IV) chloride (PtCl_4_) and dichlorodiammineplatinum (CDDP), serve as alternative viability reagents for vPCR (7). Unlike intercalating azo dyes that require photoactivation, PtCl_4_ interacts directly with nucleic acids via coordination chemistry, forming stable cross-links that prevent amplification from nonviable cells (8). To date, most of the vPCR applications employing azo dyes have focused on the detection of foodborne and waterborne pathogens, as well as probiotics (5), with the majority of studies reporting a consistent reduction in false-positive signals following dye treatment. In contrast, studies investigating PtCl_4_ remain limited, with applications primarily focused on severe acute respiratory syndrome coronavirus 2 (SARS-CoV-2) detection, where it demonstrated enhanced performance compared with azo dyes (7, 9, 10). However, Chen et al. (11) reported that PMAxx exhibited superior activity to PtCl_4_ in differentiating infectious from inactivated viruses. A common limitation across these investigations is the absence of direct comparative evaluations of different viability reagents across multiple microorganisms under standardized conditions.

In this study, we systematically compared the effects of EMA, PMAxx, and PtCl_4_ under varying treatment conditions to discriminate between viable and nonviable *Staphylococcus aureus* (*S. aureus*), *Escherichia coli* (*E. coli*), human coronavirus OC43 (HCoV-OC43; a surrogate of SARS-CoV-2), and Enterovirus A71 (EV-A71). Experiments were conducted in culture suspensions and representative real-world matrices—including nasopharyngeal swabs (NSP), milk, and vegetable wash water—employing our cost-effective, in-house-developed photoactivation device. The findings offer critical insights into the optimal conditions for vPCR implementation, thereby advancing its reliability for pathogen detection and viability assessment.

## MATERIALS AND METHODS

### Bacteria and virus culture

*S. aureus* ATCC 25923 and *E. coli* ATCC 25922 were subcultured on blood agar (BA) and incubated overnight at 37°C. HCoV-OC43 (strain VR1558), used as a surrogate for SARS-CoV-2, and EV-A71 (strain SilCRC10/TH/2011) were propagated in rhabdomyosarcoma (RD) and Vero cells, respectively, under humidified conditions at 37°C with 5% CO₂. RD cells were maintained in Dulbecco’s modified Eagle medium (DMEM), and Vero cells were maintained in minimum essential medium (MEM), each supplemented with 10% fetal bovine serum (FBS), penicillin–streptomycin, and Fungizone antibiotics (all cell-culture reagents from Gibco).

Viral titers were determined by plaque assay. Briefly, cells were seeded in 24-well plates at 2.3 × 10⁵ cells/well and incubated overnight. Monolayers were then inoculated with 100 µL of 10-fold serial virus dilutions for 1 h at 37°C in a 5% CO₂ atmosphere. After adsorption, the inoculum was removed, and the cells were overlaid with 1.56% Avicel RC-591 in 2% FBS-containing medium. Plates were incubated for 4 days (HCoV-OC43) or 3 days (EV-A71), fixed with 10% formalin in phosphate-buffered saline (PBS) for 2 h, and stained with 1% crystal violet in 20% ethanol. Plaques were counted, and viral titers expressed as plaque-forming units per milliliter (PFU/mL).

### Viability reagent preparation

EMA and PMAxx (Biotium) were employed as azo dyes. PMAxx was supplied as a ready-to-use 20 mM solution in H₂O. In contrast, EMA was provided in powdered form; 5 mg of the powder was dissolved in 1 mL of absolute ethanol or dimethyl sulfoxide (DMSO) to generate a 12 mM stock solution. All dye stock solutions were stored at −20°C in light-protected conditions. PtCl₄ (CAS No. 13454-96-1; Sigma-Aldrich) powder was dissolved in DMSO to prepare a 50 mM working stock solution, which was stored at −20°C. 

The working concentration ranges for EMA, PMAxx, and PtCl₄ were selected based on commonly applied levels summarized in previous vPCR review studies, including Canh et al. (7), which report that azo dyes are typically evaluated at 5–400 µM and platinum reagents in the low millimolar range. These literature-supported ranges were used as starting points and then refined through preliminary screening in this study.

### Nucleic acid extraction and quantification

Genomic DNA from *S. aureus* and *E. coli* was extracted using the IndiSpin Pathogen Kit (INDICAL BIOSCIENCE, Cat. No. SP54106). Viral RNA from HCoV-OC43 and EV-A71 was extracted from clarified, cell-free supernatants—obtained by centrifugation to remove intact cells and debris—using the QIAamp Viral RNA Mini Kit (QIAGEN, Cat. No. 52904). Nucleic acids were eluted in 60 µL of buffer and stored at −80°C until analysis.

Because viral RNA was isolated from supernatants rather than purified virions, a minor proportion of host-derived RNA may remain. Thus, spectrophotometric measurements reflect total recovered RNA rather than exclusively viral genomes; however, this approximation was used only for the free nucleic acid inhibition assay and does not affect the main results.

Nucleic acid concentrations (ng/µL) were converted to approximate genome copy numbers using standard molecular weight–based calculations and the assumed genome sizes for each organism: *S. aureus* (2.78 Mb), *E. coli* (4.70 Mb), HCoV-OC43 (30.7 kb), and EV-A71 (7.5 kb). Copy numbers were expressed as copies per microliter.

### Viability reagent treatment of free nucleic acids

Nucleic acid working solutions from each pathogen were adjusted to 10⁷–10⁹ copies/µL. To assess the capacity of viability reagents to suppress amplification of free nucleic acids, samples were treated with EMA at 50 or 100 µM; PMAxx at 50, 100, or 200 µM, and PtCl₄ at 50 µM or 2.5 mM.

EMA- and PMAxx-treated samples were incubated in the dark for 15 min, followed by photoactivation in an in-house device equipped with blue LED light (Thai petty patent application no. 2303000117) for 15 min to promote covalent crosslinking with nucleic acids. PtCl_4_-treated samples were incubated under light-independent conditions for 30 min at either room temperature or 4°C.

Treated and untreated nucleic acids were subsequently subjected to real-time PCR, performed as quantitative PCR (qPCR) for DNA and reverse transcription quantitative PCR (RT-qPCR) for RNA. Quantification cycle (Cq) values were recorded and compared across treatments. Cq values were further converted to copies/µL using standard curves (see File S1 at https://doi.org/10.5281/zenodo.18637100). For assays involving free nucleic acids, expressing the results as copy-number reduction provides a clearer assessment of partial or complete inhibition, particularly under conditions in which amplification is fully suppressed, thereby preventing false-positive signals.

### Viability reagent treatment of live and dead pathogens in culture suspensions

Suspensions of *S. aureus* and *E. coli* were adjusted to approximately 10⁹ CFU/100 µL in 0.85% sterile saline using McFarland standard No. 12, with final concentrations confirmed by colony enumeration on nutrient agar. HCoV-OC43 and EV-A71 were prepared at 10⁴ PFU/100 µL in ultra-distilled water (UDW). Each 100 µL suspension was divided into live and dead aliquots, with heat inactivation at 99°C for 10 min serving as the primary method for generating dead samples during treatment optimization. Bacterial inactivation was confirmed by the absence of colony growth, while viral inactivation was verified by two blind passages on RD or Vero cells without cytopathic effect.

Live and heat-inactivated aliquots were then treated with a defined panel of viability-reagent conditions, including individual azo dyes (EMA, 10–500 µM; PMAxx, 25 µM–2 mM), platinum reagent (PtCl₄, 100 µM and 2.5 mM), PMAxx–EMA combinations (1:1 at 25 µM each and 100:1 at 1 mM:10 µM), and dye formulations supplemented with membrane-permeabilizing surfactants (0.005% sodium dodecyl sulfate [SDS] or 0.01–0.05% sodium deoxycholate [DC]). For treatments containing surfactants, samples were pre-incubated with SDS or DC at 37°C for 10 min prior to dye addition to facilitate dye access to compromised cells or virions. Incubation and photoactivation conditions followed the standardized procedures described previously for nucleic acid experiments. Following treatment, nucleic acids were extracted and analyzed by real-time PCR.

Optimal treatment conditions for each organism were determined by three criteria: (i) minimal Cq shift in live controls, (ii) maximal suppression of amplification in dead controls, and (iii) the highest ΔCq between paired treated live–dead samples. After determining these optimal conditions, additional validation experiments were performed. For viruses, validation was conducted at 10² PFU/100 µL. For bacteria, validation included lower concentrations (10⁶–10⁷ CFU/100 µL) and alternative inactivation methods applied only to bacterial samples, consisting of ethanol treatment (70% ethanol, 1:1 ratio for 30 s, followed by ethanol removal by centrifugation at 8,000 rpm for 3 min and resuspension in UDW) and antibiotic inactivation using oxacillin for *S. aureus* or ampicillin for *E. coli* at 2 mg per reaction.

### Viability reagent treatment of live and dead pathogens in real-world sample matrices

*S. aureus* and *E. coli* suspensions were prepared at 10⁶ or 10⁹ CFU/100 µL and HCoV-OC43 and EV-A71 at 10² or 10⁴ PFU/100 µL. Each 100 µL suspension was divided into live and dead aliquots, with heat inactivation at 99°C for 10 min.

For bacterial matrices, 100 µL of suspension was spiked into 1 mL of milk or vegetable wash water. For viral matrices, 100 µL of suspension was added to 1 mL of nasopharyngeal viral transport medium (NSP-VTM). Aliquots of 100 µL were then either left untreated (controls) or treated under the optimal viability reagent conditions established for each pathogen. Subsequently, nucleic acids were extracted and analyzed by real-time PCR, and ΔCq values between live and dead preparations were calculated to evaluate discriminative efficacy.

### Pathogen amplification by real-time PCR

Primer sequences and amplification conditions for *S. aureus*, *E. coli*, HCoV-OC43, and EV-A71 are provided in Table S1 (12–15) at https://doi.org/10.5281/zenodo.18637100. Amplification of bacterial DNA was performed using the iTaq Universal SYBR Green Supermix (Bio-Rad, USA), while viral RNA was analyzed using the Luna Universal One-Step RT-qPCR Kit (New England Biolabs, USA). No-template controls (NTCs) were included in every run as part of standard real-time PCR quality assurance to monitor potential reagent contamination and to verify that amplification signals originated solely from sample nucleic acids. Fluorescence signals were monitored in real time using a SYBR Green detection system, and amplification curves were inspected to confirm reaction kinetics and the absence of aberrant amplification. Melting-curve analysis was subsequently performed to assess product specificity, with Tm values used to distinguish true target amplicons from nonspecific products or primer dimers.

### Digital PCR-based confirmation of complete inhibition under optimal vPCR conditions

Digital PCR was incorporated in this study not as a parallel assay for all experiments, but as a confirmatory analytical step used selectively to validate inhibition patterns observed by real-time PCR under the optimized vPCR treatment conditions. This approach allowed digital PCR to function as a high-resolution tool for verifying whether amplification signals detected or suppressed by real-time PCR corresponded to true absolute changes in target nucleic acids, without implying that digital PCR formed part of the primary comparative workflow.

Digital PCR assays were performed using the QIAcuity platform (QIAGEN, Germany) with the QIAcuity EG PCR Kit, according to the manufacturer’s protocol. Each 12 µL reaction mixture contained 4 µL of 3× QIAcuity EG PCR Master Mix, target-specific primers (final concentration at 0.2–2 µM, as optimized for each pathogen; Table S1 at https://doi.org/10.5281/zenodo.18637100), nuclease-free water, and 1–1.5 µL of template nucleic acid. The prepared reaction mixtures were loaded into nanoplates and sealed before thermal cycling.

Thermal cycling was conducted with an initial activation at 95°C for 2 min, followed by denaturation at 95°C for 15 s and annealing/extension at 60°C for 25–45 s for 37–45 cycles, depending on the target. Fluorescence signals were detected in the GREEN channel with an exposure time of 300 ms and a gain setting of 6.

NTCs were included in each run to establish the background fluorescence distribution used by the QIAcuity software to set the threshold separating positive and negative partitions. Thresholds were automatically determined by the platform’s amplitude-distribution algorithm, which identifies the trough between background and true amplification signals to ensure unbiased and consistent threshold placement. Absolute quantification (copies/µL) was subsequently computed using the software’s standard analysis pipeline.

### Statistical analysis

Data were acquired from duplicate experiments and are presented as mean ± standard deviation (SD). Statistical analysis was performed using paired *t*-tests to evaluate differences within and between groups with statistical significance defined as *P* < 0.05.

### Biosafety approval

All procedures involving pathogens classified within biological risk groups received approval from the Thammasat University Institutional Biosafety Committee (097/2566). Biosafety laboratory levels and procedural guidelines were implemented in accordance with the biological risk group of the pathogens under analysis.

## RESULTS

### Efficacy of free nucleic acid amplification inhibition after treatment

**Fig 1:**
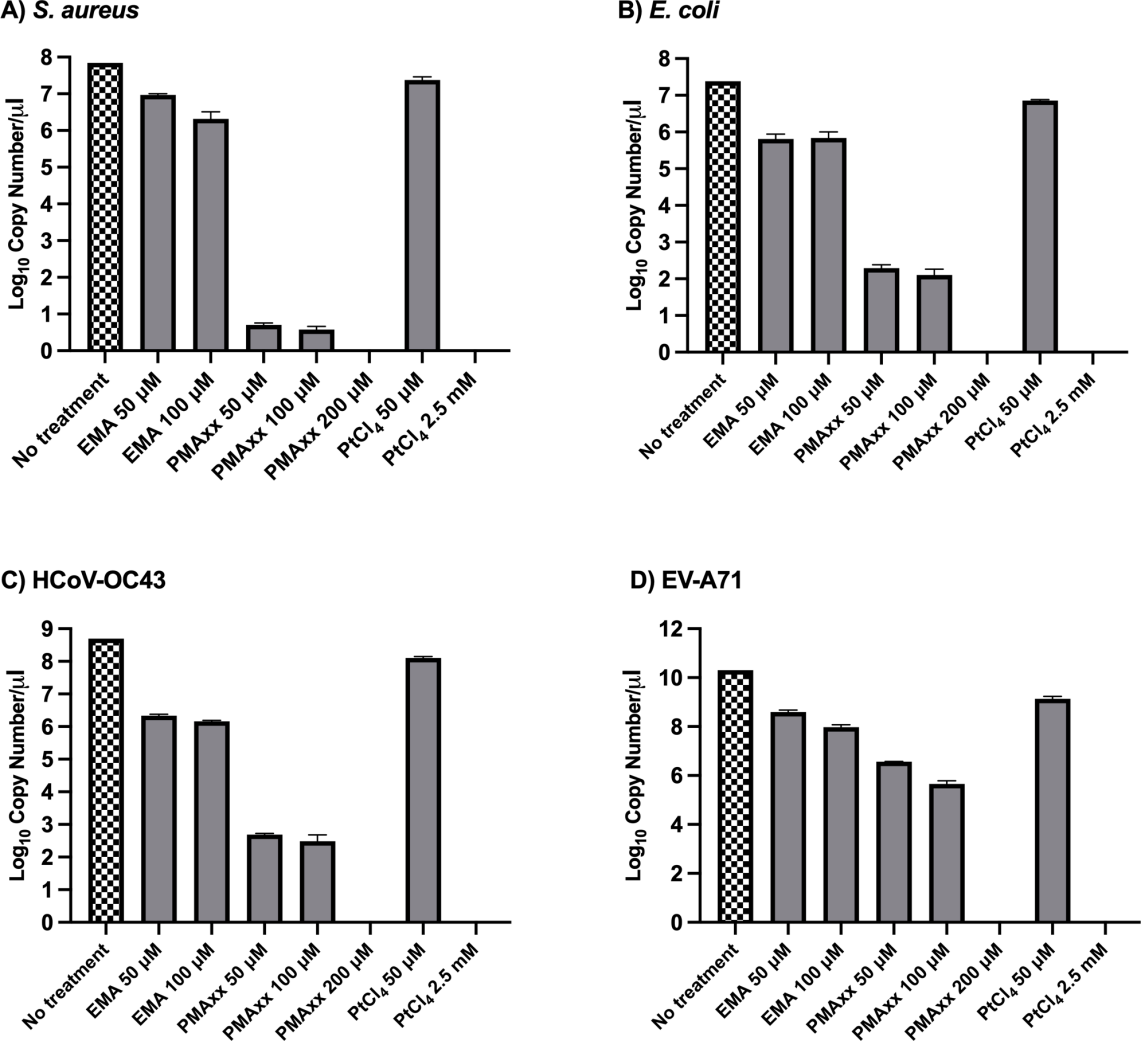
Inhibition of free nucleic acid amplification by viability reagents. (**A**) *S. aureus* DNA, (**B**) *E. coli* DNA, (**C**) HCoV-OC43 RNA, and (**D**) EV-A71 RNA at defined copy numbers per µL were used as untreated controls and subjected to amplification. Samples were then exposed to different viability-reagent treatment conditions, as indicated. The degree of amplification inhibition reflects the efficacy of each condition in preventing signals derived from free nucleic acids.

As shown in Fig. 1, the viability reagents exhibited distinct inhibitory capacities against free nucleic acids. At an identical concentration of 50 µM, PMAxx produced the greatest reduction in amplifiable nucleic acids, whereas EMA showed limited inhibition and PtCl₄ demonstrated minimal activity. Increasing PMAxx to 100 µM and 200 µM resulted in progressively greater suppression, with complete inhibition observed at 200 µM. For PtCl₄, complete inhibition was achieved only at 2.5 mM, a concentration widely reported in previous studies (9, 10) as the effective upper range for nucleic acid crosslinking. EMA did not exhibit dose-dependent improvement.

### Efficacy of discrimination between live and dead bacteria after treatment

**Fig 2:**
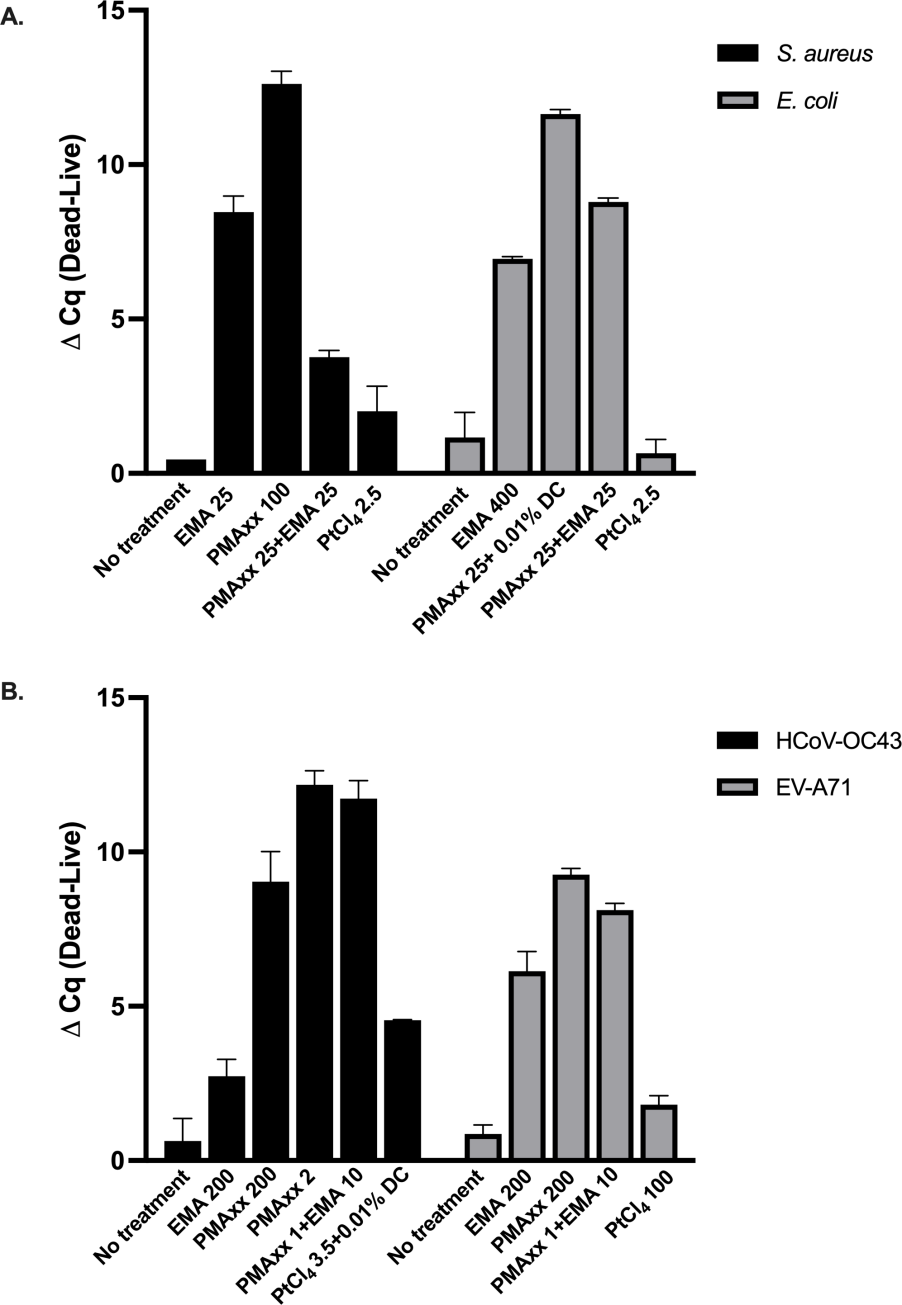
Comparison of optimal vPCR reagent conditions for discrimination between live and dead microorganisms. (**A**) *S. aureus* and *E. coli* (10^9^ CFU/100 µL) and (**B**) HCoV-OC43 and EV-A71 (10^4^ PFU/100 µL) were subjected to various vPCR reagents and conditions, and ΔCq values (dead–live) were calculated to assess discrimination efficiency. Bars represent mean ΔCq ± SD from replicate experiments. Higher ΔCq values indicate greater discriminatory capacity between live and dead bacterial cells or viral particles.

**Fig 3:**
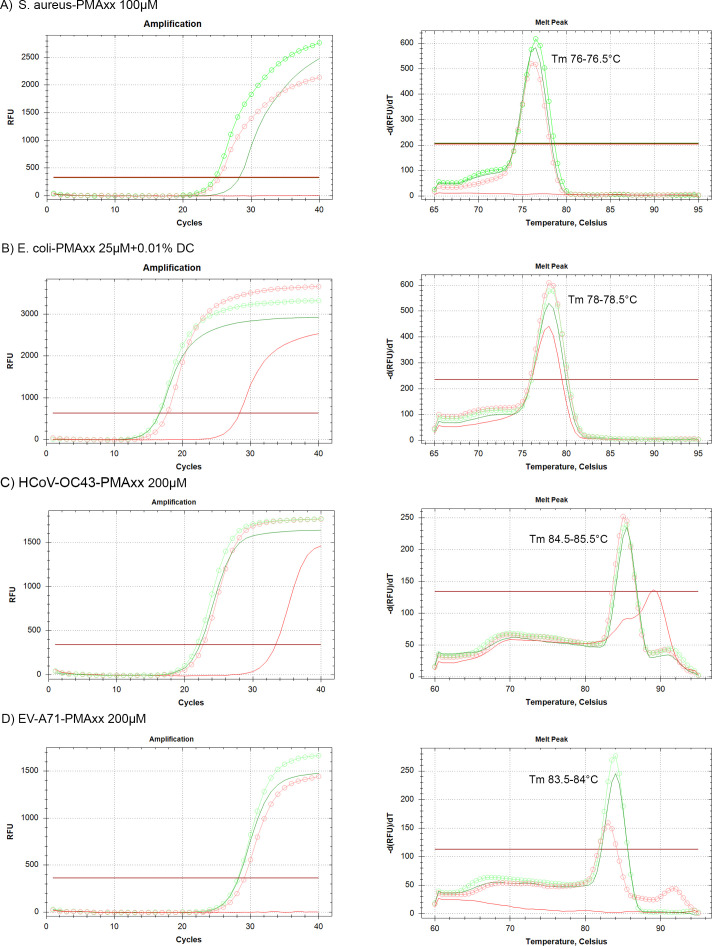
Optimal vPCR treatment conditions for discrimination of live and dead microorganisms. Amplification curves (left) and typical melting-peak profiles (right) demonstrate the most effective viability-reagent treatment condition for each organism: (**A**) *S. aureus* treated with PMAxx 100 µM at 10⁶ CFU/100 µL, (**B**) *E. coli* treated with PMAxx 25 µM plus 0.01% DC at 10⁹ CFU/100 µL, (**C**) HCoV-OC43 treated with PMAxx 200 µM at 10² PFU/100 µL, and (**D**) EV-A71 treated with PMAxx 200 µM at 10² PFU/100 µL. Green traces represent live controls (circles) and PMAxx-treated live samples (solid lines); red traces represent dead controls (circles) and PMAxx-treated dead samples (solid lines). A distinct Tm shift following PMAxx treatment is observable only for HCoV-OC43 (panel **C**).

All three viability reagents were evaluated against live and dead states of *S. aureus* and *E. coli* using varying concentrations, in combination with either 0.01% DC, 0.005% SDS, EMA, PMAxx, or combined treatments, as summarized in Table 1. At an inoculum of 10^9^ CFU/100 µL, bacteria were initially tested to identify optimal treatment conditions. Compared with untreated live controls, exposure to viability reagent exerted a more pronounced deleterious effect on live *S. aureus* than on live *E. coli*, as indicated by a larger Cq shift. At higher reagent concentrations (particularly EMA and PtCl₄), in combination with 0.005% SDS or dual azo dye treatments, this cytotoxic effect on live cells became more apparent, as illustrated in Fig. S1 at https://doi.org/10.5281/zenodo.18637100. Notably, PMAxx at 200 µM and PtCl₄ at 2.5 mM were unsuitable for intact bacterial cells, in contrast to their performance with free nucleic acids. The optimal vPCR conditions were defined as those yielding a significant ΔCq between live and heat-inactivated cells without detrimental effects on live bacteria. As shown in Fig. 2A, treatment with 100 µM PMAxx for *S. aureus* and 25 µM PMAxx combined with 0.01% DC for *E. coli* achieved the greatest ΔCq values between live and dead cells and was therefore selected as the most suitable condition. This finding was further corroborated by the amplification and melt peak profiles (Fig. 3A and B), which confirmed the effective suppression of dead-cell signals without compromising amplification from live cells. In particular, 100 µM PMAxx completely inhibited amplification of heat-inactivated *S. aureus* at 10^6^ CFU/100 µL.

**TABLE 1:** Comparative evaluation of viability-reagent treatment conditions for *Staphylococcus aureus* and *Escherichia coli^a^*

Conditions	10^9^ CFU/100 µL			10^6^ CFU/100 µL			10^7^ CFU/100 µL	
	Live	Heat	Alcohol	Live	Heat	Alcohol	Live	Antibiotics
*S. aureus*								
No treatment	16.93 ± 0.33	17.38 ± 0.33	*19.69 ± 0.21*	25.25 ± 0.16	25.68 ± 0.06	28.38 ± 0.49	21.76 ± 0.65	23.00 ± 0.06
EMA 10 μM	16.38 ± 0.10	**22.47 ± 0.18**						
EMA 25 μM	16.63 ± 0.26	**25.09 ± 0.26**	–	–	–	–	–	–
EMA 25 μM + 0.005% SDS	*23.61 ± 0.85*	27.54 ± 0.03	–	–	–	–	–	–
EMA 25 μM + 0.01% DC	*21.78 ± 0.57*	22.87 ± 0.40	–	–	–	–	–	–
EMA 50 μM	*21.87 ± 0.22*	**27.15 ± 0.79**	–	–	–	–	–	–
EMA 100 μM	*23.38 ± 0.11*	24.49 ± 0.40	–	–	–	–	–	–
PMAxx 25 μM	18.13 ± 0.12	**23.74 ± 0.09**	–	–	–	–	–	–
PMAxx 25 μM + 0.005% SDS	*25.20 ± 0.20*	27.47 ± 0.20	–	–	–	–	–	–
PMAxx 25 μM + 0.01% DC	18.96 ± 0.49	**30.09 ± 0.11**	–	–	–	–	–	–
PMAxx 50 μM	18.98 ± 0.04	**23.38 ± 0.42**	–	–	–	–	–	–
PMAxx 100 μM	19.67 ± 0.28	**32.28 ± 0.13**	**32.52 ± 0.08**	27.02 ± 0.58	**>40.00**	**33.68 ± 0.08**	23.60 ± 0.01	24.56 ± 0.30
PMAxx 100 μM + 0.01% DC	21.05 ± 1.74	29.24 ± 0.02	–	–	–	–	–	–
PMAxx 200 μM	20.56 ± 0.67	27.44 ± 0.16	–	–	–	–	–	–
PMAxx 25 μM + EMA 25 μM	*22.87 ± 0.34*	**26.63 ± 0.11**	–	–	–	–	–	–
PtCl_4_ 100 μM	17.95 ± 0.06	18.49 ± 0.30	–	-–	–	–	–	–
PtCl_4_ 2.5 mM	*23.98 ± 0.78*	25.99 ± 0.03	–	–	–	–	–	–
*E. coli*								
No treatment	16.28 ± 0.37	17.45 ± 0.45	17.63 ± 0.11	24.47 ± 0.55	25.10 ± 0.23	25.40 ± 0.03	22.27 ± 0.28	23.18 ± 0.10
EMA 50 μM	16.27 ± 0.10	**19.83 ± 0.06**	–	–	–	–	–	–
EMA 100 μM	16.20 ± 0.03	**19.83 ± 0.06**	–	–	–	–	–	–
EMA 200 μM	16.79 ± 0.10	**21.84 ± 0.11**	–	–	–	–	–	–
EMA 400 μM	16.39 ± 0.03	**23.34 ± 0.10**	–	–	–	–	–	–
EMA 400 μM + 0.005% SDS	*27.27 ± 0.08*	27.71 ± 0.01	–	–	–	–	–	–
EMA 400 μM + 0.01% DC	17.94 ± 0.08	20.67 ± 0.66	–	–	–	–	–	–
EMA 500 μM	16.48 ± 0.04	**21.73 ± 0.59**	–	–	–	–	–	–
PMAxx 25 μM	16.47 ± 0.03	**27.71 ± 0.51**	–	–	–	–	–	–
PMAxx 25 μM + 0.005% SDS	18.53 ± 0.12	**25.75 ± 0.37**	–	–	–	–	–	–
PMAxx 25 μM + 0.01% DC	16.62 ± 0.04	**28.26 ± 0.19**	**24.31 ± 0.03**	24.86 ± 0.04	**33.39 ± 0.09**	**29.66 ± 0.34**	23.37 ± 0.08	**33.78 ± 0.05**
PMAxx 50 μM	17.80 ± 0.04	**27.53 ± 0.09**	–	–	–	–	–	–
PMAxx 100 μM	19.59 ± 1.73	28.58 ± 0.35	–	–	–	–	–	–
PMAxx 25 μM + EMA 25 μM	17.01 ± 0.17	**25.80 ± 0.30**	–	–	–	–	–	–
PtCl_4_ 100 μM	16.82 ± 0.49	17.41 ± 0.3	–	–	–	–	–	–
PtCl_4_ 2.5 mM	*23.79 ± 0.32*	24.44 ± 0.77	–	–	–	–	–	–

^*a*^ Data are presented as mean ± SD. Statistical significance (*P* < 0.05) of data is indicated in italics when compared with the untreated live control and in bold when compared with the corresponding treated live condition. Cq values >40.00 were considered undetectable. “–” indicates conditions that were not tested.

**Fig 4:**
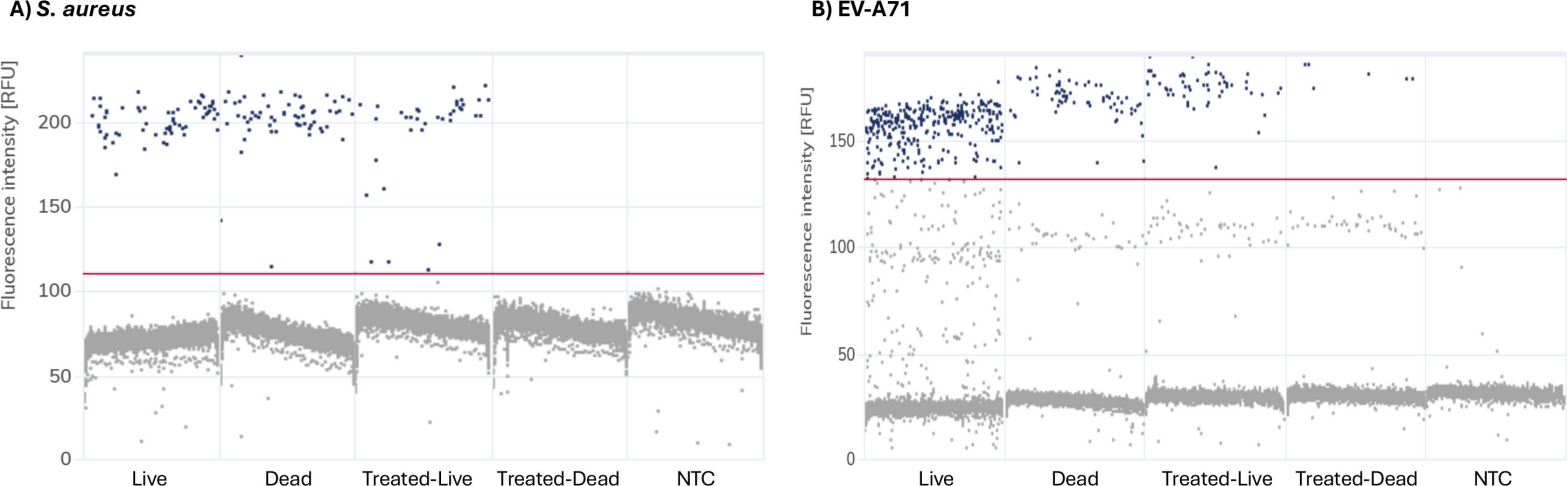
Digital PCR profiles of *Staphylococcus aureus* and Enterovirus A71 following PMAxx treatment. (**A**) Scatter plots of *S. aureus* samples analyzed using a software-generated fluorescence threshold of 109.67 RFU (red line). (**B**) Scatter plots of EV-A71 samples analyzed using a corresponding threshold of 131.31 RFU. For EV-A71, partitions clustering around ~100 RFU aligned with the amplitude distribution of the no-template control and were classified as background rather than true amplification signals. Each panel displays fluorescence amplitude (*y*-axis) versus partition number (*x*-axis), with blue and gray dots denoting positive and negative partitions, respectively. Samples are ordered from left to right as: untreated live, untreated heat-inactivated, PMAxx-treated live, PMAxx-treated heat-inactivated, and no-template control (NTC).

Digital PCR further substantiated these results, offering superior analytical sensitivity and precision compared with conventional qPCR by quantifying individual amplification events within thousands of partitions. Untreated live and heat-inactivated *S. aureus* exhibited comparable concentrations (260.73 vs 266.52 copies/µL; 62 vs 61 positive partitions), consistent with the presence of abundant free DNA in dead cells. Treatment with 100 µM PMAxx reduced, but did not abolish amplification in live cells (161.10 copies/µL; 36 positives), while completely eliminating the signal from dead cells (0 copies/µL; 0 positives), thereby confirming the selective suppression of dead-cell DNA (Fig. 4A). The enhanced sensitivity of digital PCR enabled detection of even trace residual DNA, further supporting the discriminatory capacity of the optimized vPCR conditions.

These treatments also remained effective against alcohol-inactivated cells, producing substantial Cq increases relative to untreated controls; however, heat-inactivated cells showed even greater suppression, as reflected by the higher Cq values across conditions (Table 1). In contrast, antibiotic-inactivated bacteria showed divergent responses between *S. aureus* and *E. coli*. In *E. coli*, ampicillin exposure compromised the cell wall integrity, enabling 25 µM PMAxx with 0.01% DC to penetrate and bind intracellular DNA, resulting in a significant Cq shift. In *S. aureus*, however, oxacillin inactivation largely preserved cell wall integrity, limiting dye penetration. As illustrated in Fig. S2 at https://doi.org/10.5281/zenodo.18637100, colony growth and Gram staining may support this assumption: oxacillin-inactivated *S. aureus* predominantly retained a purple Gram-positive appearance, consistent with relatively intact cell walls, whereas heat-inactivated cells stained mostly red, suggesting substantial cell wall disruption.

### Efficacy of discrimination between live and dead viruses after treatment

Similar to the bacterial experiments, both live and heat-inactivated HCoV-OC43 and EV-A71 were tested with all three viability reagents, as summarized in Table 2. Relative to untreated live controls, HCoV-OC43 was more susceptible to EMA and to higher concentrations of PtCl₄ in combination with DC surfactant, resulting in a significant Cq shift. As illustrated in Fig. 2B, PMAxx exhibited the strongest inhibitory effect on amplification of dead HCoV-OC43 among all reagents tested. No clear inhibitory effect of PtCl₄ was observed under the tested conditions. Higher concentrations of PMAxx (2 mM) or combined treatments (PMAxx 1 mM with EMA 10 µM) produced greater signal reduction in dead HCoV-OC43 than PMAxx at 200 µM. However, 200 µM PMAxx was selected for subsequent experiments as the optimal working concentration, since it provided effective inhibition of dead-virus amplification under standard assay conditions without affecting amplification efficiency or reaction consistency. At this concentration, amplification was not completely inhibited at the lower viral titer (10² PFU/100 µL); however, a Tm shift was detected in three of five replicates. The shifted peaks deviated by approximately 3–5°C from the typical Tm range (Fig. 3C), suggesting structural modification of the target nucleic acids upon PMAxx binding.

**TABLE 2:** Comparative evaluation of viability-reagent treatment conditions for HCoV-OC43 and EV-A71^*c*^

Conditions	HCoV-OV43				EV-A71			
	10^4^ PFU/100 µL		10^2^ PFU/100 µL		10^4^ PFU/100 µL		10^2^ PFU/100 µL	
	Live	Heat	Live	Heat	Live	Heat	Live	Heat
No treatment	15.63 ± 0.64	16.26 ± 0.09	21.80 ± 0.01	22.57 ± 0.11	20.83 ± 0.33	21.69 ± 0.04	28.09 ± 0.03	28.22 ± 0.03
EMA 200 μM	*21.77 ± 0.63*	24.50 ± 0.08	–	–	22.96 ± 0.69	**29.09 ± 0.04**	–	–
PMAxx 200 μM	16.29 ± 0.08	**25.33 ± 1.06**	22.07 ± 0.36	**33.38 ± 0.04^*b*^**	22.12 ± 0.11	**31.38 ± 0.10**	28.19 ± 0.06	**>40.00**
PMAxx 200 μM + 0.01% DC	16.61 ± 0.09	**25.50 ± 0.52**	–	–	22.43 ± 0.16	**31.19 ± 0.08**	–	–
PMAxx 2 mM	16.56 ± 0.08	**28.74 ± 0.54**	–	–	–	–	–	–
PMAxx 1 mM + EMA 10 μM	16.30 ± 0.16	**28.02 ± 0.75**	–	–	22.59 ± 0.12	**30.71 ± 0.09**	–	–
PtCl_4_ 100 μM	15.33 ± 0.05	16.48 ± 0.11	–	–	20.50 ± 0.04	22.31 ± 0.25	–	–
PtCl_4_ 500 μM	16.44 ± 0.33	16.77 ± 0.08	–	–	21.36 ± 0.2	21.64 ± 0.09	-	-
PtCl_4_ 2.5 mM^*a*^	17.08 ± 0.10	18.48 ± 0.16	–	–	21.22 ± 0.18	22.26 ± 0.05	–	–
PtCl_4_ 2.5 mM + 0.01% DC	17.18 ± 0.06	**20.08 ± 0.04**	–	–	21.58 ± 0.76	21.95 ± 0.13	–	–
PtCl_4_ 2.5 mM + 0.05% DC	*22.20 ± 0.21*	22.89 ± 0.72	–	–	–	–	–	–
PtCl_4_ 3.5 mM + 0.01% DC	*18.75 ± 0.30*	**23.30 ± 0.32**	–	–	–	–	–	–
PtCl_4_ 5 mM	–	–	–	–	22.62 ± 0.63	22.74 ± 0.37	–	–

^*a*^ No differences were observed when testing at 4°C, room temperature, or 37°C.

^*b*^ Tm shift was detected in 3 of 5 replicates, as shown in Fig. 3C.

^*c*^ Data are presented as mean ± SD. Statistical significance (*P* < 0.05) of data is indicated in italics when compared with the untreated live control and in bold when compared with the corresponding treated live condition. Cq values >40.00 were considered undetectable. “–” indicates conditions that were not tested.

For EV-A71, exposure of live, intact particles to the viability reagents did not alter their amplification profiles compared with the untreated control (Table 2). Among the conditions tested, PMAxx at 200 µM was identified as the most suitable, producing the largest ΔCq value (Fig. 2B). Importantly, this condition completely suppressed false-positive signals from heat-inactivated virions at 10^2^ PFU/100 µL, as shown in Fig. 3D.

Consistent with the RT-qPCR results, digital PCR analysis confirmed that PMAxx selectively reduced amplifiable targets derived from nonviable EV-A71. The untreated live sample yielded 904.4 copies/µL (309 positive partitions), followed by the heat-inactivated control (186.0 copies/µL; 67 positive partitions), the PMAxx-treated live sample (176.3 copies/µL; 57 positive partitions), and the PMAxx-treated heat-inactivated condition (17.0 copies/µL; 6 positive partitions) (Fig. 4B). The relatively high copy number observed in live control samples is likely attributable to experimental variation during cDNA synthesis or partition loading rather than a true biological difference, as previous runs using the same template source yielded comparable concentrations between untreated live and dead samples, with occasional signal fluctuation in PMAxx-treated live samples (see Fig. S3 at https://doi.org/10.5281/zenodo.18637100). These quantitative results further confirm that PMAxx effectively suppresses false-positive detection from nonviable particles while preserving amplifiable RNA from intact virions.

Likewise, none of the PtCl₄ concentrations tested produced a significant Cq difference between live and dead particles for either HCoV-OC43 or EV-A71, suggesting a limited capacity of this reagent to penetrate the viral capsid and crosslink exposed nucleic acids.

### Application of optimal vPCR conditions in simulated real-world samples

**Fig 5:**
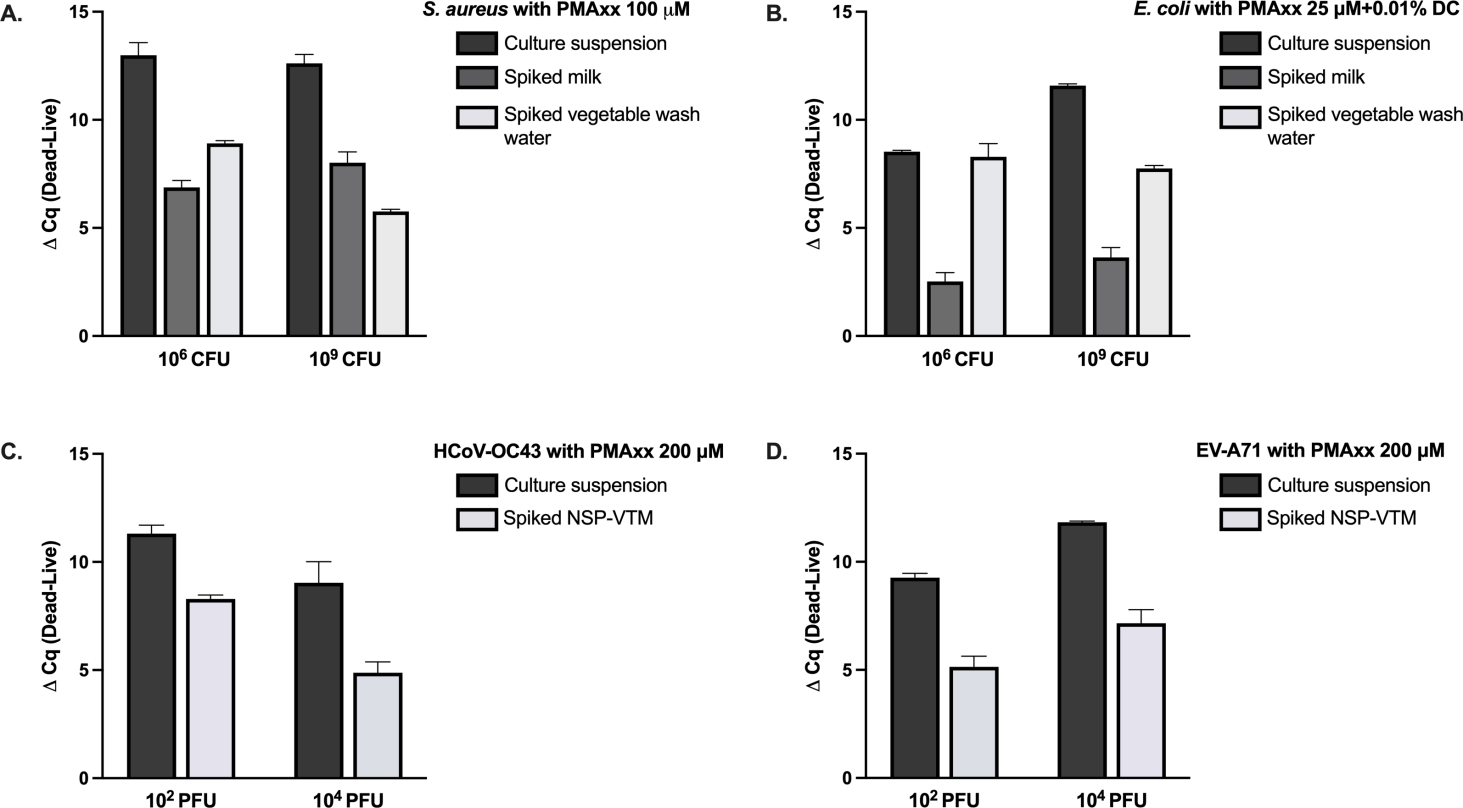
Impact of sample matrices on vPCR discrimination efficiency. The ΔCq values (dead–live; heat-inactivated vs viable) for (**A**) *S. aureus*, (**B**) *E. coli*, (**C**) HCoV-OC43, and (**D**) EV-A71 are compared between culture suspensions and samples spiked into representative matrices. Lower ΔCq values in spiked samples indicate reduced viability-discrimination efficiency attributable to matrix-associated interference. NSP, nasopharyngeal swab; VTM, viral transport medium.

In Table 3 and Fig. 5, live and heat-inactivated bacteria and viruses were artificially spiked into milk, vegetable wash water, and NSP-VTM to evaluate matrix effects on assay performance. Under the optimal vPCR treatment conditions, assays performed in milk and vegetable wash water influenced the amplification profiles of live *S. aureus*, producing a significant Cq shift; nevertheless, a clear ΔCq difference was maintained between live and heat-inactivated cells. Notably, amplification of dead *S. aureus* at 10^6^ CFU/100 µL was completely suppressed, thereby eliminating false-positive signals. In contrast, the optimal vPCR treatments did not alter the amplification profiles of live *E. coli*. However, the milk matrix impeded PMAxx penetration into compromised *E. coli* cells, whereas no such interference was observed in vegetable wash water.

**TABLE 3:** Comparative evaluation under selected optimal vPCR treatment conditions for discrimination of live and dead microorganisms in artificially spiked samples^*d*^

Conditions	Milk		Vegetable wash water		NSP-VTM	
	Live	Dead^*a*^	Live	Dead	Live	Dead
*S. aureus* 10^6^ CFU						
No treatment	26.65 ± 0.37	26.79 ± 0.35	26.00 ± 0.16	26.39 ± 0.23	–	–
PMAxx 100 μM	*30.79 ± 0.33*	**37.66 ± 0.01**	*31.10 ± 0.13*	**>40.00**	–	–
*S. aureus* 10^9^ CFU						
No treatment	17.90 ± 0.10	19.37 ± 0.29	18.21 ± 0.20	20.19 ± 0.45	–	–
PMAxx 100 μM	*21.77 ± 0.18*	**29.79 ± 0.32**	*25.76 ± 0.49*	**31.52 ± 0.39**	–	–
*E. coli* 10^6^ CFU						
No treatment	25.27 ± 0.18	25.55 ± 0.09	24.80 ± 0.05	25.25 ± 0.18	–	–
PMAxx 25 μM + 0.01% DC	26.52 ± 0.04	29.04 ± 0.45	24.93 ± 0.13	**33.22 ± 0.74**	–	–
*E. coli* 10^9^ CFU						
No treatment	17.73 ± 0.24	19.34 ± 0.16	18.59 ± 0.23	18.99 ± 0.08	–	–
PMAxx 25 μM + 0.01% DC	19.80 ± 0.01	23.44 ± 0.47	19.23 ± 0.08	**26.98 ± 0.06**	–	–
HCoV-OC43 10^2^ PFU						
No treatment	–	–	–	–	23.23 ± 0.05	24.14 ± 0.18
PMAxx 200 μM	–	–	–	–	23.83 ± 0.51	**32.12 ± 0.70^*b*^**
HCoV-OC43 10^4^ PFU						
No treatment	–	–	–	–	17.38 ± 0.16	18.38 ± 0.04
PMAxx 200 μM	–	–	–	–	19.57 ± 0.18	**24.45 ± 0.32**
EV-A71 10^2^ PFU						
No treatment	–	–	–	–	29.74 ± 0.44	30.09 ± 0.11
PMAxx 200 μM	–	–	–	–	30.44 ± 0.30	**37.59 ± 0.33^*c*^**
EV-A71 10^4^ PFU						
No treatment	–	–	–	–	22.95 ± 0.08	23.75 ± 0.13
PMAxx 200 μM	–	–	–	–	24.01 ± 0.20	**29.16 ± 0.29**

^*a*^ Dead microorganisms were obtained by heat inactivation.

^*b*^ A Tm shift associated with PMAxx treatment was observed in two of five replicates for HCoV-OC43; the Tm increased from approximately 84.5–85.5°C to 88.5–89.5°C, consistent with the profile shown in Fig. 3C.

^*c*^ A Tm shift associated with PMAxx treatment was observed across all three replicates for EV-A71; the Tm increased from approximately 83.5–84.0°C to 89.5–90.5°C, although no visually distinct melting-curve separation was present.

^*d*^ Data are presented as mean ± SD. Statistical significance (*P* < 0.05) of data is indicated in italics when compared with the untreated live control and in bold when compared with the corresponding treated live condition. Cq values >40.00 were considered undetectable. “–” indicates conditions that were not tested. NSP, nasopharyngeal swab; VTM, viral transport medium.

For HCoV-OC43 and EV-A71, application of the optimized vPCR conditions in NSP-VTM did not significantly alter the Cq values of live viruses. PMAxx retained its ability to bind viral RNA within compromised virions; however, incomplete inhibition was observed for EV-A71 at 10² PFU/100 µL, in contrast to the complete suppression obtained in culture suspensions (Table 2). This partial inhibition nevertheless yielded a clear and reproducible Cq separation between live and heat-inactivated samples. Consistent with the matrix-associated effects summarized in Table 3, discernible alterations in Tm profiles were detected for both HCoV-OC43 and EV-A71 under spiked matrix conditions. For HCoV-OC43, the numerical Tm deviations—approximately 3–5°C from the typical melting range—were comparable to the shifts observed in the culture-suspension experiment, where a subset of replicates showed similar increases (Table 3, footnote b). In contrast, EV-A71, which exhibited no visible Tm shift under culture-suspension conditions, demonstrated measurable Tm elevations across all matrix-tested replicates, with increases of approximately 5.5–7°C, consistent with the numerical changes documented in Table 3, footnote c. Taken together, these findings indicate that matrix composition can modulate dye–target interactions, thereby influencing both the extent of amplification suppression and the thermodynamic behavior of melting-curve profiles during qPCR analysis.

## DISCUSSION

The findings demonstrated that the discriminatory power of vPCR is mainly influenced by three key factors—the type of microorganism, the treatment conditions, and the inactivation method—with PMAxx consistently exhibiting the highest selectivity across both bacterial and viral targets. Our observation corroborates the systematic analysis by Leifels et al. (6), who demonstrated that PMAxx consistently provides higher viability discrimination than EMA owing to its improved membrane selectivity and photo-crosslinking efficiency.

The structural differences among *S. aureus*, *E. coli*, HCoV-OC43, and EV-A71 largely explain the observed variations in reagent efficacy. Gram-positive *S. aureus* possesses a thick peptidoglycan layer that limits dye penetration even after inactivation, whereas *E. coli*, as a Gram-negative bacterium, is more permeable following membrane damage. In contrast, HCoV-OC43 is an enveloped RNA virus whose lipid envelope readily undergoes disruption upon heating or surfactant exposure, facilitating PMAxx entry into the damaged capsid. EV-A71, a non-enveloped enterovirus, exhibited the most stable structure, reflecting the intrinsic robustness of picornavirus capsids. These structural differences substantiate that pathogen composition—cell wall, membrane, or envelope—represents a primary determinant of vPCR performance.

Importantly, this study was not designed to define a universal vPCR treatment applicable to all tested pathogens. Instead, the bacterial and viral assays were designed to comparatively evaluate the performance of major reagent classes under representative conditions. Given the fundamental structural and permeability differences among Gram-positive bacteria, Gram-negative bacteria, enveloped viruses, and non-enveloped viruses, species-specific variation in optimal dye concentration and detergent assistance is expected and well documented in vPCR literature. Accordingly, the treatment sets examined here reflect a targeted, literature-informed refinement rather than an exhaustive factorial optimization, which would be experimentally impractical and scientifically unnecessary for the comparative scope of this work.

Several previous studies have demonstrated that incorporating surfactants can enhance vPCR performance by facilitating dye penetration through compromised cell membranes or disrupted viral envelopes (16, 17). In the present study, DC at 0.01% exhibited superior performance compared with SDS, indicating that the efficiency of surfactant-assisted vPCR strongly depends on both surfactant type and concentration. Consistent with our earlier report (18), excessive concentrations of nonionic surfactants, such as Triton X-100 and Tween 20 (1–3%), can adversely affect live viral particles, resulting in signal attenuation. These findings underscore the importance of carefully tailoring reagent composition and matrix conditions to maximize viability discrimination while minimizing cytotoxicity and false-negative outcomes in vPCR assays.

This study highlights that the method of pathogen inactivation critically influences the discriminatory power of vPCR, as the degree of structural compromise directly determines the ability of viability reagents to penetrate target cells or virions. Pathogen inactivation that preserves overall structural integrity—such as UV exposure—has been widely recognized to result in poor dye penetration and subsequent failure of viability discrimination (19, 20). In contrast, heat treatment induced greater structural disruption than alcohol or antibiotic exposure, thereby facilitating deeper penetration of PMAxx and significantly reducing false-positive amplification signals (21). Notably, PMAxx penetration was ineffective in oxacillin-treated *S. aureus*, whereas it succeeded in ampicillin-treated *E. coli*. This difference may be attributed to the upregulation of cell wall synthesis–related genes or the expression of penicillin-binding protein 2a (PBP2a) in *S. aureus*, which confers resistance to osmotic lysis and promotes a non-lytic survival state (22, 23). Collectively, these findings suggest that the mechanism of pathogen inactivation should be considered when interpreting vPCR results or designing applications for real-world diagnostic or environmental settings.

Compared with earlier vPCR studies that employed qPCR alone, this work integrated digital PCR as a novel platform for absolute quantification of viable pathogens. Previous studies have primarily focused on bacteria such as *Listeria*, *Salmonella*, *E. coli*, and various probiotic species (5), as well as on viruses, including foodborne enteric viruses and the recently emergent SARS-CoV-2 (7). To our knowledge, no prior study has evaluated vPCR using digital PCR across both bacterial and enteric or respiratory viral systems within a unified comparative framework. A small number of reports have applied viability dPCR to selected bacterial species (24–26). Among these, studies by Yang et al. (25) and Li and Bae (26) directly compared qPCR with digital PCR, demonstrating that dPCR offers improved quantitative resolution and superior sensitivity for detecting viability-associated differences, particularly at low target concentrations or in VBNC states. In contrast, Dhar et al. (24) employed dPCR without a qPCR comparison, reflecting the increasing shift toward dPCR as a preferred high-precision platform for viability assessments. Our findings are consistent with these observations: digital PCR provided absolute quantification that corroborated the complete suppression of amplification in nonviable preparations under optimized vPCR conditions, while maintaining quantifiable signal in viable samples. The modest variability observed in EV-A71 results likely reflects differences in cDNA synthesis efficiency or partition loading, as noted in Fig. S3 at https://doi.org/10.5281/zenodo.18637100, rather than genuine biological variation.

Contrary to earlier reports in which PtCl₄ successfully suppressed signals from heat-inactivated SARS-CoV-2 RNA (9, 10), our study found no inhibitory effect of PtCl₄ against live-tested HCoV-OC43, EV-A71, or bacterial samples. Notably, those studies evaluated only inactivated viral preparations, without direct comparison to live counterparts. Our findings are consistent with the observations of Chen et al. (11), who demonstrated that PMAxx better discriminated infectious from inactivated viruses in heat-processed berries. These discrepancies underscore that assays incorporating infectious viral controls provide a more accurate assessment of viability discrimination.

This study advances current understanding by incorporating artificial simulated real-world matrices, such as milk, vegetable wash water, and viral transport medium, thereby providing practical insights into matrix-associated interferences that frequently limit assay sensitivity (6). Matrix-interference experiments were included because viability PCR assays used in real diagnostic or environmental settings must operate within complex sample types rather than simple laboratory buffers. Constituents commonly present in food and environmental matrices can impede dye penetration or alter PCR amplification efficiency, thereby reducing assay discrimination. Consistent with this rationale, the matrix-associated effects highlighted in Table 3 demonstrate that vPCR performance is strongly influenced by the composition of the suspension medium. Both milk and vegetable wash water reduced assay discrimination, with noticeable attenuation of amplification efficiency, particularly for *E. coli*, likely due to inhibitory organic components that interfere with dye accessibility. For *S. aureus*, these matrices also produced a significant upward shift in Cq values for live cells, indicating partial inhibition of amplification even when membrane integrity was preserved. Because the present study aimed to evaluate reagent robustness under representative, unmodified matrix conditions, no sample-conditioning steps (such as centrifugation and resuspension into a defined buffer) were applied. Nonetheless, such conditioning steps may be advantageous during future assay standardization to minimize matrix-associated interference in routine workflows. Likewise, practical implementation of vPCR typically requires a defined control structure—including a no-treatment control, a live-cell control, and a heat-inactivated control—to verify dye selectivity; in some applications, a DNA-only control may further assist in confirming complete suppression of free nucleic acids. While the present comparative design did not aim to standardize an operational workflow, these methodological considerations remain important for translating vPCR into practical diagnostic or environmental testing pipelines.

Overall, the results demonstrate that PMAxx maintains reliable viability discrimination across bacterial and viral systems, even in the presence of challenging sample matrices. These observations support its practical utility in vPCR workflows and highlight its potential for broader application. Future studies should extend this evaluation to additional pathogen types and to authentic clinical or environmental samples, where performance may be influenced by more complex biological and physicochemical variables. Complementary use of digital PCR in such settings may further enhance quantitative confidence and help refine vPCR protocols for routine diagnostic or environmental surveillance purposes.

### Conclusion

This study compared EMA, PtCl₄, and PMAxx for viability PCR across bacterial and viral targets. PMAxx showed the most reliable discrimination, effectively suppressing nonviable signals at 25–200 µM using a standard treatment of 15-min dark incubation and 15-min photoactivation, while preserving viable amplification. Digital PCR further confirmed the complete suppression of dead-cell signals under these optimized conditions. Although matrix components reduced overall assay efficiency, PMAxx maintained robust performance, supporting its applicability for viability-based pathogen detection in both controlled and complex sample types.

## Data Availability

The supplemental materials and associated raw data are publicly available in the Zenodo repository (https://doi.org/10.5281/zenodo.18637100).
